# Spreading of healthy mood in adolescent social networks

**DOI:** 10.1098/rspb.2015.1180

**Published:** 2015-08-22

**Authors:** E. M. Hill, F. E. Griffiths, T. House

**Affiliations:** 1 Centre for Complexity Science and Warwick Infectious Disease Epidemiology Research Centre, University of Warwick, Coventry CV4 7AL, UK; 2 Warwick Medical School, University of Warwick, Coventry CV4 7AL, UK; 3 School of Mathematics, University of Manchester, Manchester M13 9PL, UK

**Keywords:** social contagion, emotional contagion, depression

## Abstract

Depression is a major public health concern worldwide. There is evidence that social support and befriending influence mental health, and an improved understanding of the social processes that drive depression has the potential to bring significant public health benefits. We investigate transmission of mood on a social network of adolescents, allowing flexibility in our model by making no prior assumption as to whether it is low mood or healthy mood that spreads. Here, we show that while depression does not spread, healthy mood among friends is associated with significantly reduced risk of developing and increased chance of recovering from depression. We found that this spreading of healthy mood can be captured using a non-linear complex contagion model. Having sufficient friends with healthy mood can halve the probability of developing, or double the probability of recovering from, depression over a 6–12-month period on an adolescent social network. Our results suggest that promotion of friendship between adolescents can reduce both incidence and prevalence of depression.

## 1. Introduction

Depression and other mood disorders are major and growing contributors to mortality and morbidity worldwide [1]*.* These mood disorders are widespread, with the World Health Organization estimating that, globally, there are currently more than 350 million people affected by depression [2]. There is evidence that social support is important for the mental well-being of adolescents [3] and that befriending can have a positive effect on mental health [4]. Recent experiments suggest that people's expression of negative or positive emotions is influenced by the level of negative or positive news from their friends and associates [5]. An improved understanding of the social processes that drive the epidemiology of these diseases therefore has the potential to bring highly significant public health benefits.

It is now very common to model infectious diseases as spreading processes on networks [6]. This approach is increasingly applied to behaviours (e.g. those related to infectious risk [7]) and non-infectious diseases that are linked to behaviours that can spread socially (e.g. obesity and smoking [8,9]).

Previous work relating to spreading of depression on social networks has generally made at least one of the following key assumptions: (i) low mood and/or depression spreads like an infectious agent; (ii) healthy mood (non-depression) does not spread like an infectious agent; (iii) the information to distinguish between transmission and no-transmission models can be found in differences in static network measures such as clustering of disease [10–12], or in coarse population-level measures such as web-search over time [13]. Here, we allow more flexibility in our model by making no prior assumption as to whether it is low mood or healthy mood that spreads. In addition, we use the dynamical behaviour of mood over time, allowing us to distinguish directly between transmission and no transmission.

## 2. Material and methods

### (a) The data

We consider data from the in-home interview survey of the Add Health study [14], which recorded adolescents' in-school friends in addition to their CES-D (Center for Epidemiologic Studies Depression Scale) scores [15]. This was used to classify individuals as either having depressive symptoms (low mood) or not being depressed (healthy mood) according to the score cut-off associated with a clinical diagnosis of depression [16]. We took data from two time points (waves) 6 to 12 months apart, from students in saturated schools (all students in a saturated school were selected to have an in-home interview, eliminating selection bias). To be included in our study sample, for both time points the student must have provided complete answers to all the CES-D survey-related questions and be the least restricted in the number of school friends they were allowed to give (each student was either allowed to list up to five male and five female friends, or limited to listing a maximum of one male and one female friend, with students in the latter group not considered for inclusion in our study sample).

### (b) Model construction, fitting and selection

We model depression status as a discrete-time Markov chain, where each individual *i* at time *t* has state *X_i_*(*t*), taking either the value *D* for depressive symptoms or *N* for not depressed. This model is specified by two probabilities: the probability *p* = Pr[*X_i_*(*t* + 1) = *D* | *X_i_*(*t*) = *N*] of becoming depressed, and the probability *q* = Pr[*X_i_*(*t* + 1) = *N* | *X_i_*(*t*) = *D*] of recovering from depression. Following Centola and Macy [17,18], we considered a model in which these probabilities depend on the number of friends of an individual who have value *N* or *D*, with this dependence taking the form of an S-shaped function. These models are referred to as *N* transmits and *D* transmits, respectively. We then fit this model with the Add Health data moving from wave 1 to wave 2, and compare with the no-transmission model that the probabilities do not depend on the moods of an individual's friends. Parameter values for our transmission and no-transmission models were found using maximum-likelihood estimation (MLE) by minimizing the negative log-likelihood –log(*L*) with respect to *p* and *q* using the MATLAB fmincon() function. Confidence intervals were obtained through calculation of the Hessian matrix at the MLE parameters and use of standard asymptotic formulae. Appendix A outlines the construction of the likelihood functions used in the fitting process. Competing models were assessed using the Akaike information criterion (AIC) [19].

### (c) Simulation outline

A discrete-time Monte Carlo simulation of the no-transmission model and *N*-transmits model was performed on a directed network of named friends constructed from the 3084 individuals in the dataset satisfying our inclusion criteria at the first time point (wave 1). We took 10^5^ independent samples from the stationary distribution for each model to calculate model quantities of interest including uncertainty. We assessed uncertainty in the observed quantities through bootstrapping. The Bonferroni method was used to account for multiple testing of statistically significant differences between models and observed data [20]. To further test the goodness-of-fit of our chosen transmission model, residual errors were analysed via a parametric bootstrap approach (see the electronic supplementary material).

## 3. Results

### (a) Fitted parameter values

#### (i) No-transmission models

We obtained the no-transmission deterioration model for transitioning from healthy mood to low mood within a year
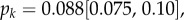
and the no-transmission recovery model for transitioning from low mood to healthy mood within a year,
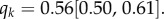


#### (ii) *N*-transmits models

We obtained the *N*-transmits deterioration model for transitioning from healthy mood to low mood within a year,
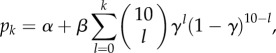
with 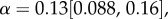 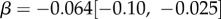 and 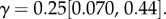 Note that here and elsewhere numbers such as 10 appear as the limits in the data on number of friends; *k* is the number of friends in the transmitting state, and the parameters estimated are a simple way to parametrize a discrete sigmoidal function as suggested by a complex contagion model. The *N*-transmits recovery model, for transitioning from low mood to healthy mood within a year, was
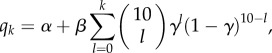
with 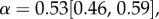 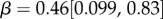 and 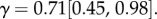


#### (iii) *D*-transmits models

We obtained the *D*-transmits deterioration model for transitioning from healthy mood to low mood within a year,
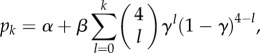
with 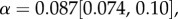 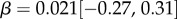 and 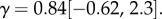 The *D*-transmits recovery model, for transitioning from low mood to healthy mood within a year,
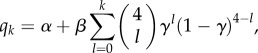
with 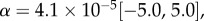 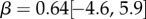 and 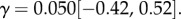


### (b) Model comparisons

**Figure 1.**
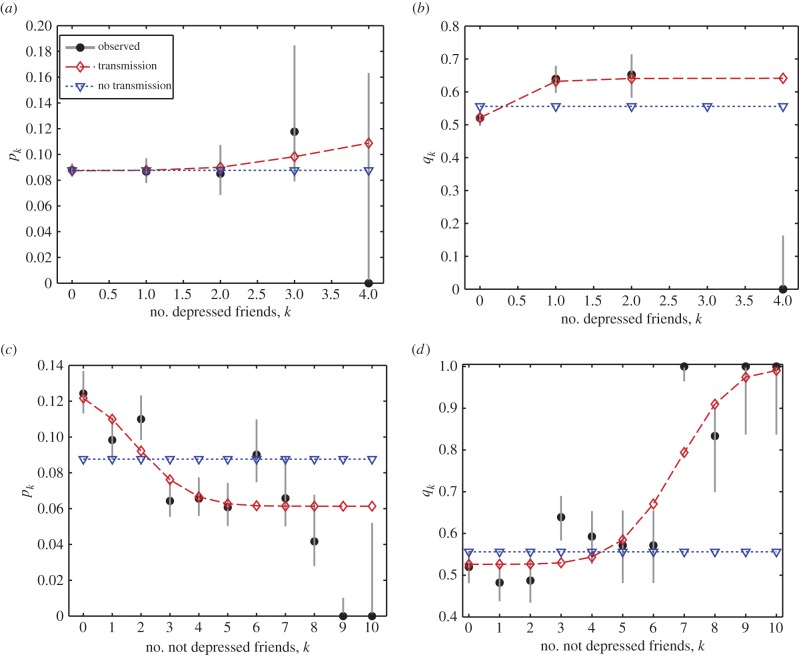
Dynamical behaviour of depression status between samples as a function of *N* friends or *D* friends for observed data (and 50% CI), transmission and no-transmission models. Uncertainty in the observed values was quantified using Jeffreys intervals [21]. The ΔAIC value is calculated by subtracting the no-transmission AIC value from the transmission AIC value. (*a*) Probability of transitioning from healthy mood to low mood against number of *D* friends—transmission is not preferred to no transmission (ΔAIC ≈ −4). (*b*) Probability of recovering from low mood against number of *D* friends—transmission is not preferred to no transmission (ΔAIC ≈ −0.9). (*c*) Probability of transitioning from healthy mood to low mood against number of *N* friends—transmission is preferred to no transmission (ΔAIC ≈ 8.4). (*d*) Probability of recovering from low mood against number of *N* friends—transmission is preferred to no transmission (ΔAIC ≈ 4.5).

Figure 1 shows the results of fitting the different models to the *n* = 2194 data points given by our inclusion criteria. For the dependence of probabilities *p* and *q* on the number of friends with depressive symptoms (no-transmission model against *D*-transmits model), AIC values showed the no-transmission model was the preferred choice (figure 1*a*,*b*). When considering the no-transmission model against the *N*-transmits model, the *N*-transmits model was the preferred choice in both cases (figure 1*c*,*d*).

### (c) Simulation analysis

**Figure 2.**
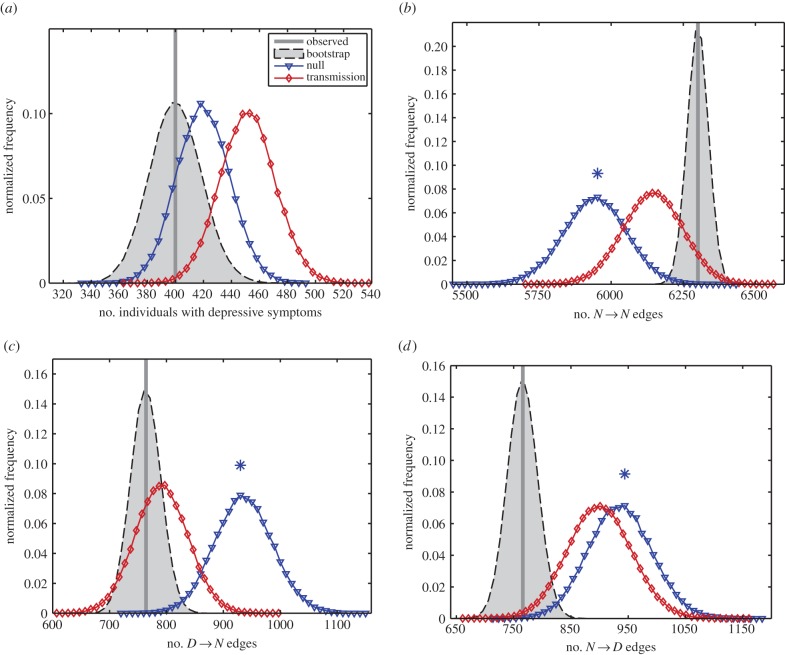
Static summary statistics for the stationary distributions of the models versus real data. Asterisks above a plot denote a significant statistical difference at the 5% level, corresponding to *p* < 0.01 using the Bonferroni method to account for multiple testing. (*a*) Prevalence of individuals with depressive symptoms—observed data could be plausibly generated by both transmission (*p* = 0.058) and no-transmission (*p* = 0.41) models. (*b*) Number of *N* → *N* edges—observed data could be plausibly generated by the transmission model (*p* = 0.15) but not by the no-transmission model (*p* = 0.0014). (*c*) Number of *D* → *N* edges—observed data could be plausibly generated by the transmission model (*p* = 0.54) but not by the no-transmission model (*p* = 0.0035). (*d*) Number of *N* → *D* edges—observed data could be plausibly generated by the transmission model (*p* = 0.027) but not by the no-transmission model (*p* = 0.0067). The fifth test and plot is for *D* → *D* edges (electronic supplementary material, figure S1). See Appendix B for the *p*-value calculation method, and electronic supplementary material, figure S2, for stratification by number of contacts.

Comparing *D* prevalence and edge type summary statistics to those obtained for the observed data, there were significant differences between the no-transmission model and data, while the transmission model (with the probability dependent on the number of *N* friends) had no statistically significant differences (figure 2). In particular, the *N* → *N*, *N* → *D* and *D* → *N* edge statistics (where we write A → B for an individual in state A naming an individual in state B as a friend) were found to be statistically significantly different between the no-transmission model and the data (figure 2*b*–*d*). We also assessed goodness-of-fit and parameter identifiability through simulation, giving extra confidence to our results (see the electronic supplementary material).

## 4. Discussion

**Figure 3.**
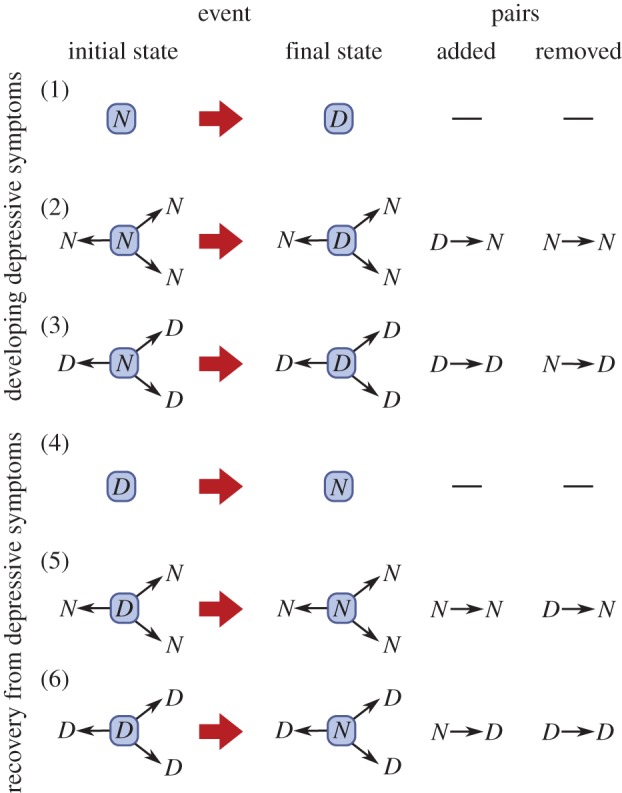
Pictorial representation of the possible events in our model: developing or recovering from depressive symptoms; in the absence of friends, with friends with healthy mood, or with depressed friends. The changes in pairs produced are also shown.

A major benefit of the dynamical approach that we have taken is that it avoids the problems of confounding that have been controversial in other studies of social contagion [12]. Figure 3 shows the model schematically, to provide intuitive insight into why this is the case.

In this model, there is transmission of *D* if the probability of event (3) happening (given the initial state) is bigger than the probability of event (1) happening (given the initial state): Pr(event (3)) > Pr(event (1)). There is also transmission of *D* if Pr(event (6)) < Pr(event (4)). We did not find evidence for transmission of *D* based on this criterion, as shown in figure 1*a*,*b*. Such transmission would also be expected to lead to more *D* → *D* pairs and fewer *D* → *N* and *N* → *D* pairs than a null model. This pattern was not observed (see figure 2; electronic supplementary material, figure S1).

There is transmission of *N* in our model if Pr(event (2)) < Pr(event (1)) and also if Pr(event (5)) > Pr(event (4)). We found statistically significant evidence for transmission of *N* on the basis of this criterion, as seen in figure 1*c*,*d*. Such transmission would also be expected to lead to more *N* → *N* pairs and fewer *D* → *N* and *N* → *D* pairs than a null model. This pattern was observed (figure 2).

Suppose that there is homophily (similar individuals naming each other as friends) at work in the social network, either in terms of depressive symptoms, or a latent variable that is correlated with depressive symptoms. This will tend to increase the number of *D* → *D* or *N* → *N* pairs in the absence of any transmission effect, meaning that these tests (shown in figure 2) can be confounded by homophily. When working with two waves of data, however, such homophily will simply lead to fewer individuals in the initial states associated with events (3) and (5) than events (2) and (6), but in our approach we fit to the probability of moving to a final state given an initial state. This means that since there are still sufficient data to find a statistically significant effect, homophily cannot confound the results shown in figure 1. Our verbal argument here can be made in a more mathematically precise manner, as shown in the electronic supplementary material.

In summary, we have shown the epidemiological impact of such mood transmission in a large adolescent population, giving statistically significant evidence for spreading of healthy mood, but not for spreading of depressive symptoms. Once discovered, this behaviour is in fact plausible through a number of mechanisms. Depression has been associated with social withdrawal [22], and so depressed individuals would be expected to exert less social influence than adolescents with healthy mood. However, each individual may need sufficient exposure to others with a healthy mood in order to stay well, or become well if depressed. In support of this, there is evidence from psychology of mechanisms by which mood is transmitted between people. Automatic transmission of mood between people has been demonstrated [23]. Unconscious mimicry enhances social rapport [24], and those feeling positive towards the person with whom they are interacting socially are more likely to mimic, and so build rapport [25], and thus the opportunity for transmission of healthy mood. People who are (or have a tendency to be) depressed are less able to maintain a positive outlook from moment to moment [26], a deficit potentially compensated by interaction with healthy friends.

The static network measures provide indirect evidence of spreading of healthy mood through analysis of clustering, which shows that the no-transmission model is significantly different from the observed data, while the data and *N* transmits model are in agreement. Such clustering, while supportive of a transmission effect, can have other causes, and so we recommend that future empirical work measure changes in mood over time where possible.

Our results offer implications for improving adolescent mood. In particular, they suggest the hypothesis that enabling networks of friendship between adolescents has the potential to reduce both incidence and prevalence of depression. Our complex contagion model suggests that adolescents with five or more healthy friends have half the probability of becoming depressed over a 6–12-month period compared with adolescents with no healthy friends, and that adolescents with 10 healthy friends have double the probability of recovering from depressive symptoms over a 6–12-month period compared with adolescents with three healthy friends. If such an effect were demonstrated in an intervention study, this would massively outperform existing interventions.

## Supplementary Material

Supplementary Material

## Data accessibility

The data analysed in this study were obtained on licence from Add Health. Requests for restricted-use data access can be made to Add Health at http://www.cpc.unc.edu/projects/addhealth/data/restricteduse/add-health-restricted-use-data-contract-1 or addhealth@unc.edu.

## Appendix A. Likelihood functions

The following likelihood function was constructed for the development of the ‘depressive symptoms’ scenario, with respect to either the number of not-depressed friends or number of friends with depressive symptoms:

where *y_k_* was the number of respondents with *k* not-depressed friends (friends with depressive symptoms) who were classified as not depressed at the first time point and having depressive symptoms at the second time point. *N_k_* was the total number of respondents classified as not depressed at the first time point with *k* not-depressed friends (friends with depressive symptoms). An equivalent likelihood function was constructed for the ‘recovery from depressive symptoms’ scenario:

with *y_k_* corresponding to the number of respondents with *k* not-depressed friends (friends with depressive symptoms) who were classified as having depressive symptoms at the first time point and not depressed at the second time point. *D_k_* was the total number of respondents classified as having depressive symptoms at the first time point with *k* not-depressed friends (friends with depressive symptoms).

## Appendix B. *p*-value calculation

To calculate the *p*-values in figure 2 and electronic supplementary material, figure S1, required comparison of Monte Carlo simulation output with uncertain data, and so we used the expression

where *π_x_* is the density of the value *x* in the bootstrap sample from data, and *E_x_* is the empirical cumulative distribution function of the simulation output.
