# Supplementary material for: Spreading of healthy mood in adolescent social networks
Source: Proc Biol Sci. 2015 Aug 22;282(1813):20151180. doi: 10.1098/rspb.2015.1180 (PMC4632626; doi:10.1098/rspb.2015.1180)
Supplement: Supplementary Material [file rspb20151180supp1.pdf]

Spreading of healthy mood in adolescent social networks: Supplementary Material

E M Hill F E Griffiths T House

Contents

1 Formal definitions **1**

2 Simulation methods **2**

3 Prevalence of $D \to D$ pairs **3**

4 Dependence on degree **3**

5 Goodness-of-fit **4**
5.1 Residual error calculation . . . 4
5.2 Simulations . . . 4
5.3 Results . . . 4

6 Analysis of confounding **5**
6.1 Setup . . . 5
6.2 Homophily model . . . 6
6.3 Transmission model . . . 6
6.4 Other models . . . 7

7 Parameter Identifiability **7**

1 Formal definitions

Throughout, we write N for healthy mood and D for depression. Letters $A, B, \ldots \in \{N, D\}$ and overlining is used as follows:

$$\overline{A} = \begin{cases} N & \text{if } A = D \ , \\ D & \text{if } A = N \ . \end{cases} \tag{1}$$

Let individuals be labelled with indices $i, j, \ldots \in \{1, \ldots, n\}$. At (discrete) time t individual i has state $X_i^t \in \{N, D\}$. These are connected on a network with adjacency matrix $\mathbf{G}$ with elements

$$G_{ij} = \begin{cases} 1 & \text{if individual } i \text{ named } j \text{ as a friend,} \\ 0 & \text{otherwise.} \end{cases} \tag{2}$$

Our general N-transmits model is of a discrete-time Markov chain $\mathbf{X}^t = (X_i^t)$ with transition

probabilities

$$\begin{aligned}\mathbb{P}\big[X_i^{t+1} = D \big| X_i^t = N\big] &= p_{\sum_j G_{ij} \mathrm{I}\{X_j^t = N\}} \ , \\ \mathbb{P}\big[X_i^{t+1} = N \big| X_i^t = D\big] &= q_{\sum_j G_{ij} \mathrm{I}\{X_j^t = N\}} \ , \end{aligned} \tag{3}$$

where I is an indicator function

$$\mathrm{I}\{\omega\} = \begin{cases} 1 & \text{if } \omega \text{ is true,} \\ 0 & \text{otherwise.} \end{cases} \tag{4}$$

In the no transmission model, p_k and q_k are independent of k and in the D-transmits model we exchange D for N on the right-hand side of (3). We will often be interested in just two timepoints, which we will write as t and $t+1$ in general.

2 Simulation methods

The Markov chain defined by (3) can be simulated using standard Monte Carlo methods. Note that since we consider a situation where

$$0 < p_k, q_k < 1, \forall k, \tag{5}$$

then we can get from any state to any other in a finite number of steps (the chain is irreducible) and the expected time to return to any state will be finite (all states are non-null persistent) and hence by e.g. Theorems (6.4.3) and (6.4.17) of Grimmett and Stirzaker (2001), there will be a unique stationary distribution π that describes the behaviour of the chain at large times.

To sample from this distribution, we perform discrete-time Monte Carlo simulation of the models that are specified by values of p_k, q_k on a directed network of named friends constructed from the $n = 3084$ individuals in the dataset satisfying our inclusion criteria at the first time point (wave 1). Depending on the simulated output required, we took 10^4 time-separated samples of either network pairs at a single time point or temporally adjacent node-level state transitions from the stationary distribution for each model.

3 Prevalence of $D \to D$ pairs

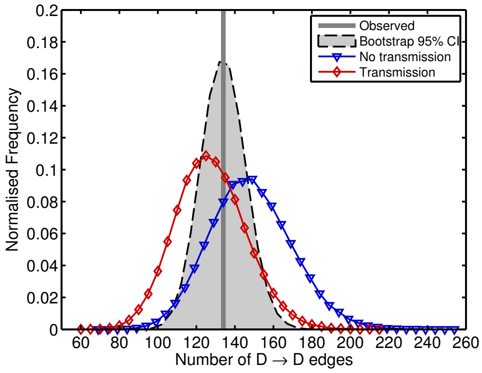

Fig. S1: Number of $D \to D$ edges for the stationary distributions of the models versus real data.Asterisks above a plot denote a significant statistical difference at the 5% level, corresponding to $p < 0.01$ using the Bonferroni method to account for multiple testing. Observed data could be plausibly generated by both transmission ($p = 0.59$) and no transmission ($p = 0.60$) models.

4 Dependence on degree

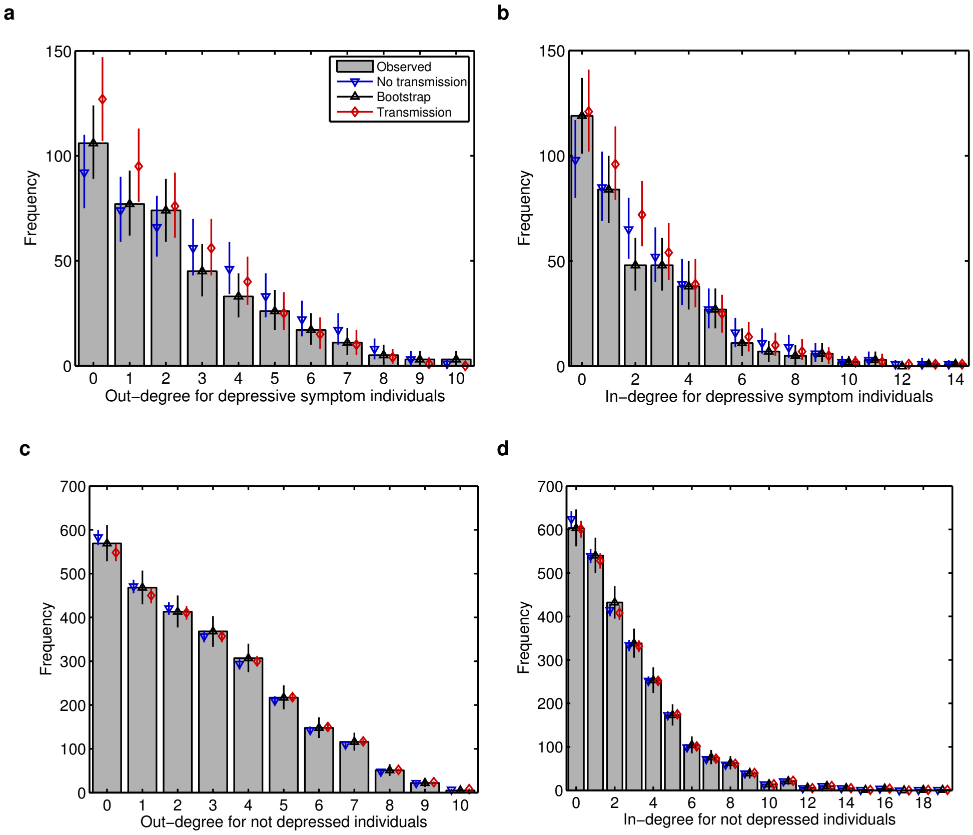

Fig. S2: Features of the transmission and no transmission models as compared to data. (a) out-degree of individuals with depressive symptoms; (b) in-degree of individuals with depressive symptoms; (c) out-degree of not depressed individuals; (d) in-degree of not depressed individuals.

5 Goodness-of-fit

5.1 Residual error calculation

For logistic regression, a standard approach to assessing goodness-of-fit is the Hosmer-Lemeshow (HL) test, which is based on the distribution of residual errors (Hosmer and Lemeshow, 2005) – i.e. the differences between the observed and the model values. Our model is not a standard regression, and so we test goodness-of-fit in a similar manner to the HL test but with assumptions more appropriate for our model. In particular, we define a residual error function stratified by number of friends,

$$\mathcal{E}_A = \left(\sum_{k=0}^{10} \left(Y_k^{A\to\overline{A}} - X_k^{A\to\overline{A}}(\theta) \right)^2 \right)^{1/2} , \tag{6}$$

where $Y_k^{A\to\overline{A}}$ is the observed number of state transitions from A to $\overline{A}$ of individuals with k friends in state N, and $X_k^{A\to\overline{A}}(\theta)$ is the modelled number of such events given parameters θ; explicitly

$$\begin{aligned} Y_k^{A\to B} &= \sum_i \mathrm{I}\{\textstyle\sum_j G_{ij} = k\}\mathrm{I}\{Y_i^{t+1} = B\}\mathrm{I}\{Y_i^t = A\} , \\ X_k^{N\to D}(\theta) &= p_k(\theta) \sum_i \mathrm{I}\{\textstyle\sum_j G_{ij} = k\}\mathrm{I}\{X_i^t = N\} , \\ X_k^{D\to N}(\theta) &= q_k(\theta) \sum_i \mathrm{I}\{\textstyle\sum_j G_{ij} = k\}\mathrm{I}\{X_i^t = D\} . \end{aligned} \tag{7}$$

The quantity $\mathcal{E}_A$ is positive definite and will tend to zero for a model that perfectly captures the data.

5.2 Simulations

The distribution for $\mathcal{E}_A$ is not analytically available, and so we use a parametric bootstrap approach, simulating from the model once it has been fitted to observed data by maximum-likelihood estimation (MLE), giving MLE parameter estimate $\hat{\theta}$.

We performed simulations as detailed in §2 above, extracting the proportion of individuals who recover from depressive symptoms / gain depressive symptoms within a year, dependent on the number of friends in different states they had at the initial time point. This sampling process was repeated many times as for other bootstrap methods to obtain an accurate estimate of the distributions of $\mathcal{E}_D$ and $\mathcal{E}_N$.

5.3 Results

Fig. S3 shows observed and simulated residual error values $\mathcal{E}_D$ and $\mathcal{E}_N$, together with associated p values. Note that in this case, the simple residual error summary statistic does not have any asymptotic properties that suggest it should be used in model selection in the way that AIC is, therefore special attention should not be paid to any particular threshold of p value; rather, a larger p value simply denotes a better fit. These results therefore support our broad conclusion that N-transmits should be preferred to no transmission.

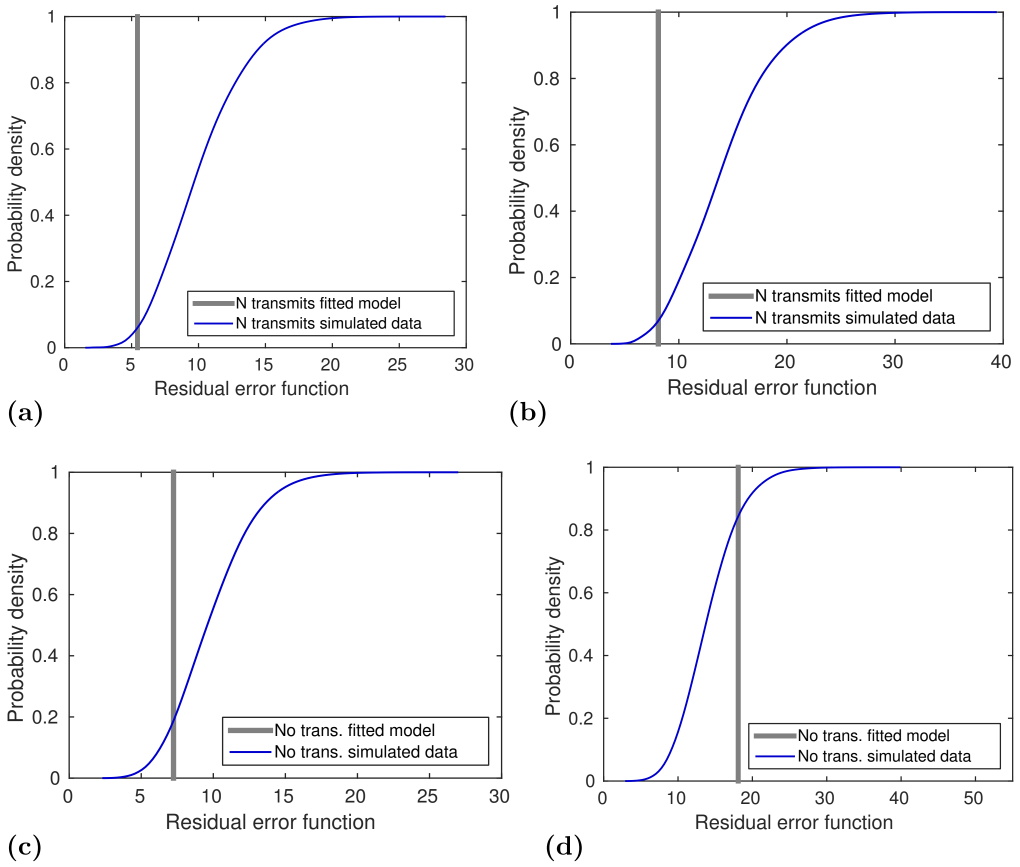

Fig. S3: Cumulative distribution functions for residual errors, obtained via parametric bootstrapping, versus the observed data residual error. (a) N-transmits recovery model residual error function cumulative distribution ($p = 0.94$); (b) N-transmits deterioration model residual error function cumulative distribution ($p = 0.94$); (c) no transmission recovery model residual error function cumulative distribution ($p = 0.82$); (d) no transmission deterioration model residual error function cumulative distribution ($p = 0.15$).

6 Analysis of confounding

6.1 Setup

Our aim here is to state in mathematical language what is meant by transmission of mood, how confounding is possible and not possible. We will do this using pairwise model notation, and will write $[A]$ for the number of nodes of state A, $[A \to B]$ for the number of individuals in state A naming an individual in state B, at a given time point that we will normally omit; formally

$$[A] = \sum_i \mathrm{I}\{X_i^t = A\} \ , \qquad [A \to B] = \sum_{i,j} \mathrm{I}\{X_i^t = A\}\mathrm{I}\{G_{ij} = 1\}\mathrm{I}\{X_j^t = B\} \ . \tag{8}$$

We are going to consider how to calculate relevant quantities for both a transmission model and a model with homophily relating to some unobserved property ξ.

6.2 Homophily model

Suppose we have a property (or vector of properties) that individuals have, for example age, socio-economic status, or spatial location. We label these properties with ξ and write $[\xi]$ for the number of nodes that are of ξ etc.

Now consider a relatively general model in which the probability of changing state if in state A and with property ξ is ρ^A_ξ. We can then write down equilibrium values for the expected number of pairs under the stationary distribution π, which are

$$\mathbb{E}_\pi[A \to B] = \sum_{\xi,\xi'} \mathbb{E}_\pi[\xi \to \xi'] \frac{\rho^{\overline{A}}_\xi}{\rho^{\overline{A}}_\xi + \rho^A_\xi} \frac{\rho^{\overline{B}}_{\xi'}}{\rho^{\overline{B}}_{\xi'} + \rho^B_{\xi'}} \ . \tag{9}$$

It is clear that by tuning the propensity of different property types to name each other as friends, and the transition probabilities, arbitrary pair structures can be created. But for the transitions, we have that at equilibrium

$$\mathbb{E}_\pi \left[X^{A\to A}_k\right] = \sum_\xi \mathbb{E}_\pi[A_\xi](1-\rho^A_\xi) \ , \qquad \mathbb{E}_\pi \left[X^{A\to \overline{A}}_k\right] = \sum_\xi \mathbb{E}_\pi[A_\xi]\rho^A_\xi \ . \tag{10}$$

These do not depend on k. Overall, therefore, this model cannot be falsified from observations of numbers of pairs $[A \to B]$, but can be falsified from observations of transitions stratified by k, $Y^{A\to B}_k$.

6.3 Transmission model

In general, therefore

$$\begin{aligned} X^{N\to D}_k &\sim \mathrm{Bin}\Big(p_k,\ \sum_i \mathrm{I}\{X^t_i = N\}\mathrm{I}\{(\textstyle\sum_j G_{ij}\mathrm{I}\{X^t_j = N\}) = k\}\Big) \ , \\ X^{D\to N}_k &\sim \mathrm{Bin}\Big(q_k,\ \sum_i \mathrm{I}\{X^t_i = D\}\mathrm{I}\{(\textstyle\sum_j G_{ij}\mathrm{I}\{X^t_j = N\}) = k\}\Big) \ . \end{aligned} \tag{11}$$

This means that given the freedom to choose p_k, q_k for a given network configuration, it is possible to tune the expected values of these transitions to whatever value is required. The probabilities assigned to different network configurations under the invariant distribution π do not in general have an analytic closed form solution. In the event where p_k and q_k do not depend on k, then equations of the form (10) will hold where every individual has the same property ξ.

In the event where the population has size n and there are on average m friends per individual, note that basic combinatorial considerations give that

$$m[N] = [N \to N] + [N \to D] \ , \quad m[D] = [D \to N] + [D \to D] \ , \quad n = [N] + [D] \ , \tag{12}$$

meaning that there are only three independent parameters: $[N]$; $[N \to D]$; and $[D \to N]$. Now suppose that p_k is monotone decreasing with k and if q_k is monotone increasing with k, this will lead to fewer $[D \to N]$ pairs than equations of the form (10) would suggest due to transmission of N.

6.4 Other models

It is, of course, possible to combine elements of the transmission and homophily models in various ways. We take the philosophical position that anything more complex than the homophily model above will constitute a *mechanism* for the phenomenon of social contagion rather than an alternative to it.

7 Parameter Identifiability

We now turn to the question of how accurately model parameters can be inferred from data. To do this, we performed simulations as in §2 above. Each set of simulated data was then fitted to the same model that it had been generated from using MLE.

Fig. S4 shows histogram outlines of inferred N-transmits model parameter values. These N-transmits simulated data fitted models were compared to the observed data N-transmits fitted model values. A high level of identifiability was observed for each parameter.

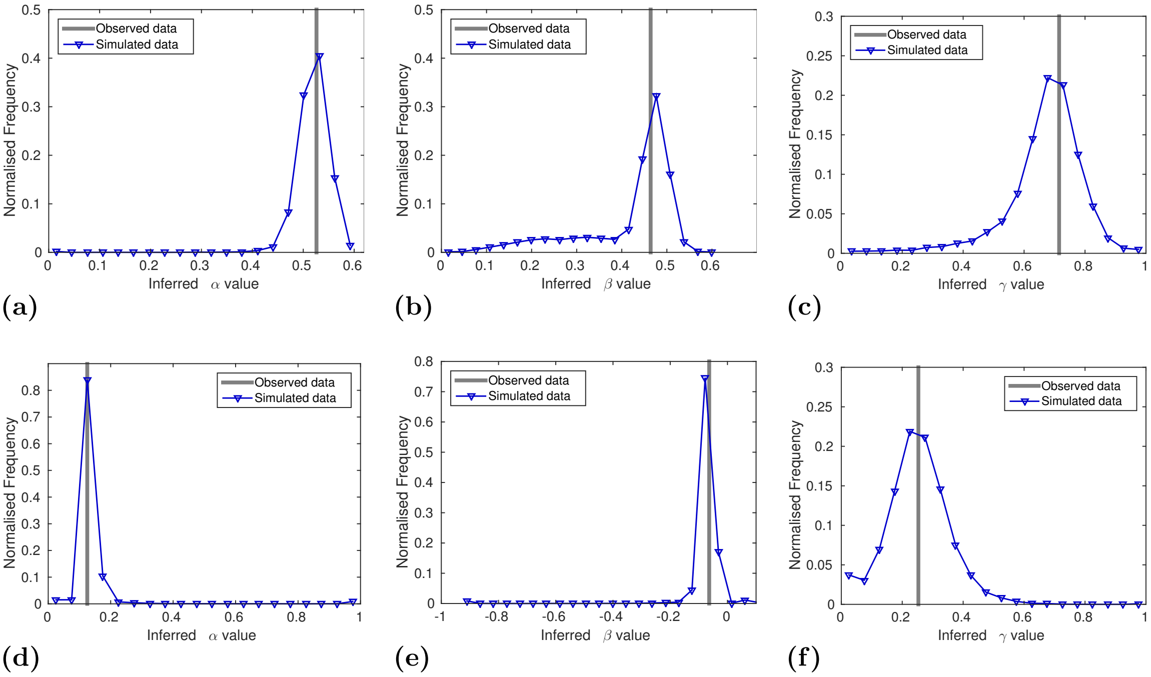

(a) (b) (c) (d) (e) (f)

Fig. S4: Normalised frequency of inferred N-transmits model parameters from 10^4 simulated samples for (a,b,c) transition from N to D and (d,e,f) transition from D to N versus chosen model values when fitted to the observed data. (a,d) α; (b,e) β; (c,f) γ.

References

G. Grimmett and D. Stirzaker. *Probability and Random Processes.* OUP, Oxford, 2001.

David W. Hosmer and Stanley Lemeshow. *Applied Logistic Regression.* John Wiley & Sons, Inc., 2005.
